# Merit, equity, and administrative competence in the retirement of the objective structured clinical exam

**DOI:** 10.1177/10398562241233995

**Published:** 2024-02-25

**Authors:** Andrew Amos, Michael Weightman, Edward Miller

**Affiliations:** 104397 Townsville, QLD; 1066 1065 Adelaide, SA; 1415 Auckland, NZ

Dear Editor,

We welcome the opportunity to correct a few of the errors in Schuwirth et al.’s^1^ response to our analysis of their decision to retire the OSCE from the psychiatric training programme.^2^ Given their prominent roles in fundamentally changing psychiatric training in Australasia, it is concerning that they do not appear to understand our extension of their own work.

They state that we confused predictive with construct validity, but our paper does not mention either concept. Following Schuwirth’s comparison of the OSCE with the alternative assessment pathway (AAP),^3^ we assumed a normal distribution of scores relative to a cut-off representing competence. We agree that this is an unsophisticated way to analyse the assessment of competence, but it was Schuwirth’s decision to do so, not ours. We extended Schuwirth’s analysis by varying the simplifying assumptions and examining the consequences. As we followed Schuwirth in assuming a gold-standard cut-off representing competence, the question of validity simply does not arise.

We agree there are more meaningful ways to model the impact of the move from OSCE to AAP and would have analysed such a model had one been available. Our main point was the need to release such a model before fundamentally changing the method of evaluating competence in the Fellowship pathway. We take the authors’ failure to mention any such modelling as confirmation that it does not exist. We note that the authors did not address another key point from our paper: that the substantially higher pass rates under the AAP suggest a lower standard of assessment.

We also agree it is regrettable if our analysis has cast doubt on the competence of recently ‘fellowed’ psychiatrists. However, we find it curious that Schuwirth et al.^1^ imply the fault is ours for raising the question, rather than theirs, for not having released modelling necessary to justify the change in practice. If the modelling has been done, it should be publicly released, so it can be publicly debated. Until it is done, we remain sceptical about the value of low-stakes examination for establishing clinical competence.

One final point of agreement is that our analysis focused on the statistical assessment of competence to the exclusion of other considerations such as equity. We justify this focus on the basis that the stress caused by high-stakes exams may be regrettable, but a training programme that cannot distinguish competent from pre-competent trainees would be disastrous. We question whether the authors’ focus on issues like the former has obscured their view of the latter.
